# Impact of Manufacturing Variability and Washing on Embroidery Textile Sensors

**DOI:** 10.3390/s18113824

**Published:** 2018-11-08

**Authors:** Marc Martinez-Estrada, Bahareh Moradi, Raúl Fernández-Garcia, Ignacio Gil

**Affiliations:** Department of Electronic Engineering, Universitat Politècnica de Catalunya, 08222 Terrassa, Spain; marc.martinez.estrada@upc.edu (M.M.-E.); bahareh.moradi@upc.edu (B.M.); ignasi.gil@upc.edu (I.G.)

**Keywords:** sensor, e-textile, embroidery, moisture, conductive yarn

## Abstract

In this work, an embroidered textile moisture sensor is presented. The sensor is based on a capacitive interdigitated structure embroidered on a cotton substrate with an embroidery conductor yarn composed of 99% pure silver plated nylon yarn 140/17 dtex. In order to evaluate the sensor sensitivity, the impedance of the sensor has been measured by means of a impedance meter (LCR) from 20 Hz to 20 kHz in a climatic chamber with a sweep of the relative humidity from 25% to 65% at 20 °C. The experimental results show a clear and controllable dependence of the sensor impedance with the relative humidity. Moreover, the reproducibility of the sensor performance subject to the manufacturing process variability and washing process is also evaluated. The results show that the manufacturing variability introduces a moisture measurement error up to 4%. The washing process impact on the sensor behavior after applying the first washing cycle implies a sensitivity reduction higher than 14%. Despite these effects, the textile sensor keeps its functionality and can be reused in standard conditions. Therefore, these properties point out the usefulness of the proposed sensor to develop wearable applications within the health and fitness scope including when the user needs to have a life cycle longer than one-time use.

## 1. Introduction

Textiles have been revealed as a natural and convenient substrate choice in the development of wearable electronic applications due to the fact that humans have been covering our body with fabrics for thousands of years [1]. This fact, together with the rapid miniaturization of electronic components and the development of new materials is allowing for the integration of electronic functionalities on fabrics, using well known textile manufacturing techniques, such as weaving knitting, embroidery, etc. [2]. Among the techniques, embroidery has been revealed as the most effective technique to implement wearable electronics due to the availability of the manufacturing technology and the flexibility of the technologies to make different geometries and layouts over the textiles [3]. Among the different embroidery e-textile applications, in the last years, a great effort has been focused on designing new e-textile sensors that are included in garments [4]. Many of the studies are focused on fields such as health monitoring [5], physical training [6], emergency rescue service, and law-enforcement [7]. 

Previous literature mainly reports on single use sensors. In order to guarantee the long term functionality of these devices, two topics should be addressed: the variability of the electrical behavior with the manufacturing process and the functionality of the involved e-textiles after washing cycles. In this sense, only a few works can be found in the literature focused on the electrical behavior of e-textile after washing cycles [8,9,10]. These previous publications suggest that the electrical behavior of e-textile is modified after several washing cycles. In order to delve in depth in this topic, a capacitive embroidered textile moisture sensor is presented and a full characterization of its response was carried out, taking into account the manufacturing variability and the washing cycles.

The remainder of the paper is organized as follows. Section 2 describes the Material and methods where the textile sensor layout is defined and the measurement set-up as well as the washing cycle’s procedures are described. In Section 3 the experimental results are shown and discussed. Finally, in Section 4 the conclusions are summarized.

## 2. Materials and Methods

**Figure 1:**
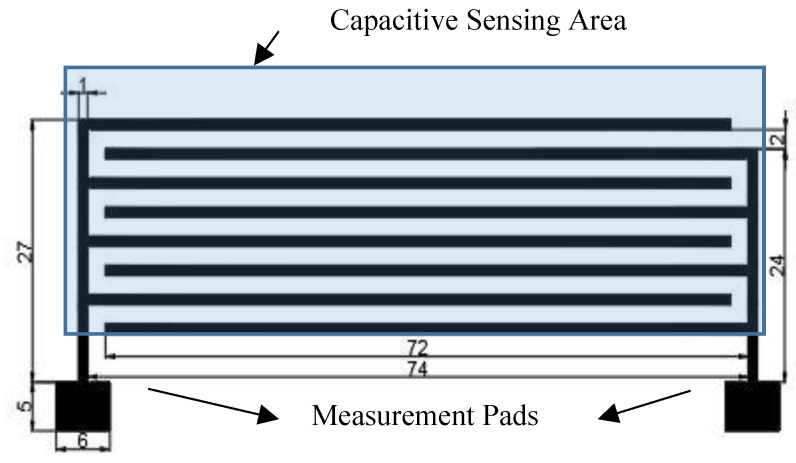
Layout and dimension detail of the proposed moisture sensor (in mm). The bottom squares correspond to the characterization pads, and the capacitive sensing area corresponds to the interdigitated area.

The proposed moisture sensor is based on a capacitive embroidered interdigitated structure whose dimensions are depicted in Figure 1. In this structure, the capacitive sensor performance depends on the geometry (i.e., number of fingers, size, and distant between fingers) and the substrate material permittivity. If a hygroscope material is used as a substrate, the permittivity of the substrate will be modified under the presence of water molecules. This mechanism gives the sensing capability to the proposed devices.

A commercial Shieldex 117/17 dtex 2-ply was chosen as a conductive yarn in order to embroider the interdigitated structures on a high hygroscope substrate. Specifically, a cotton substrate with a thickness (h) of 0.43 mm was chosen. A Singer Futura XL-550 embroidery machine (Singer Corporation, La Vergne, TN, USA) with a satin fill stitch pattern was selected in order to achieve a homogeneous yarn distribution over the sensor surface. With this configuration, the embroidery machine dimension resolution was 100 µm.

**Figure 2:**
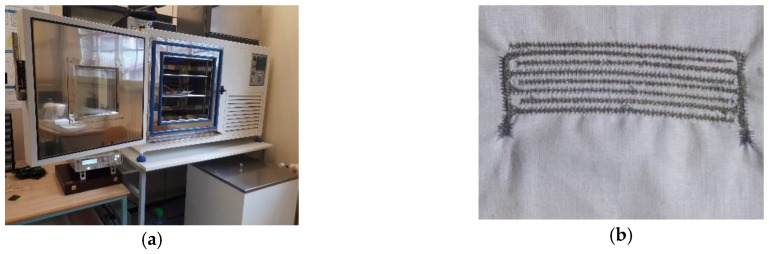
Image of the experimental setup. (**a**) CCK-25/48 Dycometal (**b**) Embroidered capacitive sensor.

In order to characterize the sensor behavior, the device was tested in a CCK-25/48 Dycometal climatic chamber (Dycomental Equipos S.L., Viladecans, Spain), and the sensor impedance was measured by means of an external Rohde & Schwarz HM8118 LCR meter (Rohde & Schwarz, Munich, Germany). The LCR and sensor connection was done through a feed cable hole on the climatic chamber chassis. An image of the experimental setup and the embroidered sensor are shown in Figure 2. 

The sensor impedance was measured in a frequency range from 20 Hz to 20 kHz in a 25% to 65% relative humidity environment, meanwhile, the temperature remained constant at 20 °C. In order to guarantee and analyze the reproducibility, ten different samples were characterized and analyzed at 200 Hz, and the average and standard deviation was used as a figure of merit. 

**Figure 3:**
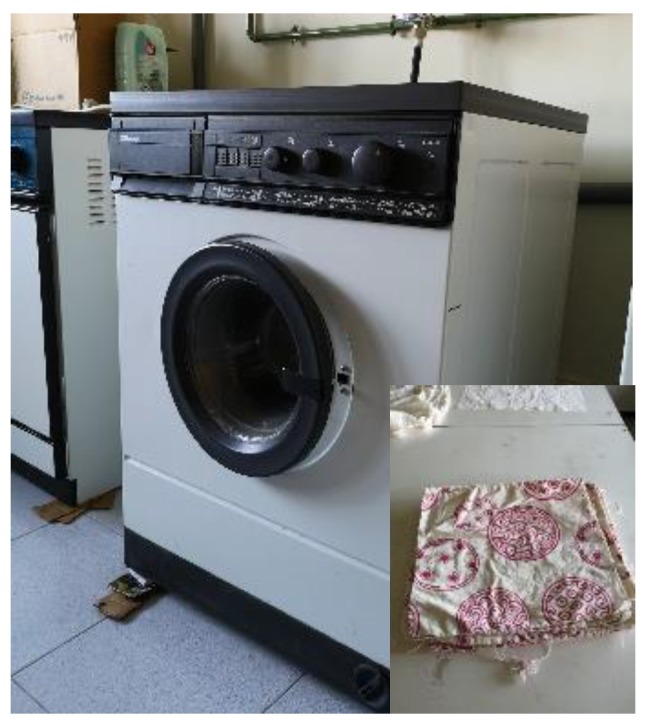
Image of the experimental Balay T5609 washing machine and the support fabric for washing cycles (inset).

Finally, in order to evaluate the impact of washing cycles on the electrical behavior, the electrical impedance was measured before and after putting the samples into the washing cycles. For this process, the selected soap and the washing machine were used according to the standard requirements defined on the UNE-EN ISO 6330:2012. A neutral ECE-Color Detergent ISO 105-C06 soap (Testgewebe Gmbh, Brüggen, Germany) was used and 1 kg of support fabric was used in every wash (Figure 3). A washing machine (Balay T5609, BSH Electrodomesticos, Zaragoza, Spain) was configured at 1000 rpm and temperature of 40 °C, and 1% by weight of soap (i.e., 10 g) was introduced in the washing machine.

## 3. Results and Discussion

**Figure 4:**
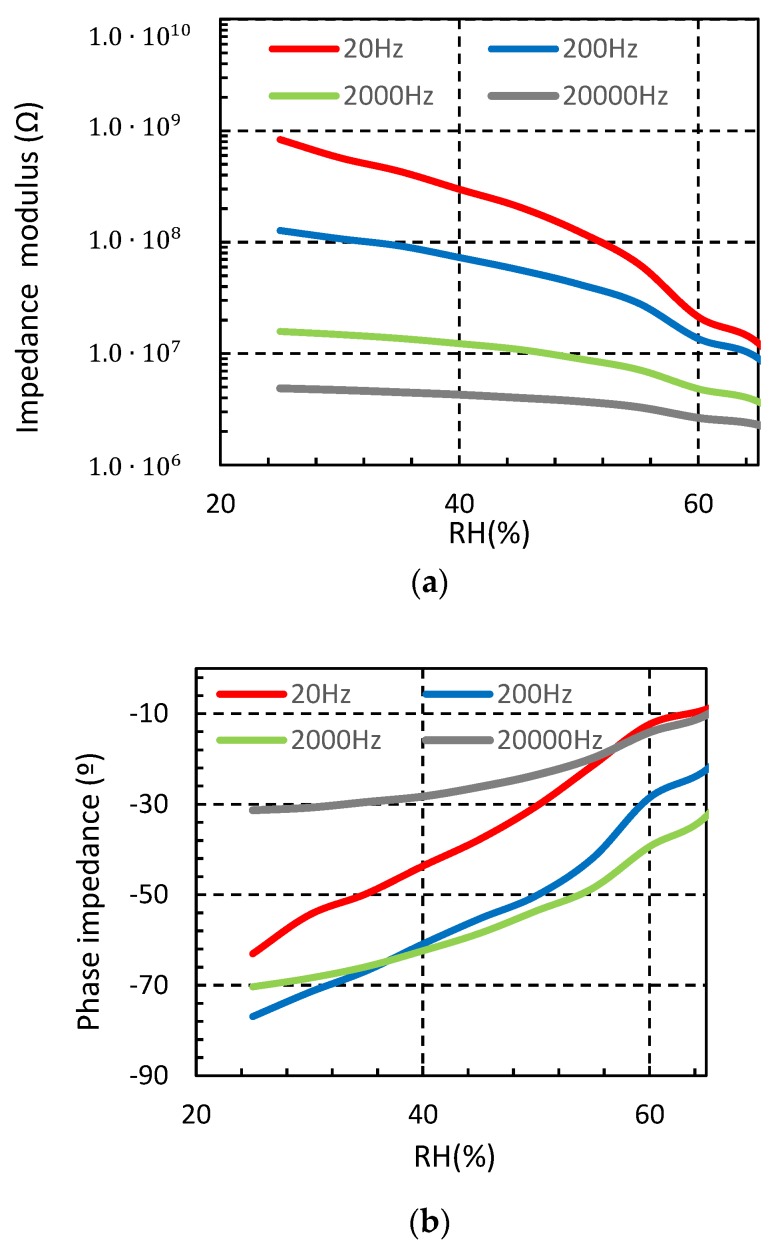
Measured sensor impedance from 25% to 65% relative humidity (RH) at different frequencies (T = 20 °C) (**a**) impedance modulus (**b**) impedance phase.

Figure 4 shows the measured sensor impedances when the moisture is increased from 25% to 65% for four different test frequencies. It is observed that the impedance module of the sensor is reduced when the environmental moisture increases. This fact confirms the functionality of the proposed structure as a moisture sensor. The measured phase impedance of the sensor is negative in all the studied frequency ranges, denoting that for low relative humidity, the sensor has a capacitive behavior, as expected. However, for higher relative humidity concentrations, the sensor tends to be resistive. The reason of this behavior is the hydrophilic property of the cotton. Indeed, when the relative humidity increases, the cotton substrate absorbs water, and the electrical permittivity of the substrate increases. As a result, the impedance of the sensor is reduced. In particular, for the 200 Hz test signal, the sensor impedance module decreases from 127 MΩ to 9.08 MΩ when the moisture increases from 25% to 65%. For the same moisture range, the phase impedance increases from −76.92° to −22.38°.

### 3.1. Manufacturing Variability

Once the functionality of the proposed sensor to measure the ambient moisture was demonstrated, the reproducibility of this sensor was evaluated in order to know the impact of manufacturing variability on its performance. In this analysis, the previous sensor capacitor structure was used, and the electrical impedance of ten samples was measured from 25% to 65% relative humidity (RH) at 200 Hz with a 95% confidence interval.

**Figure 5:**
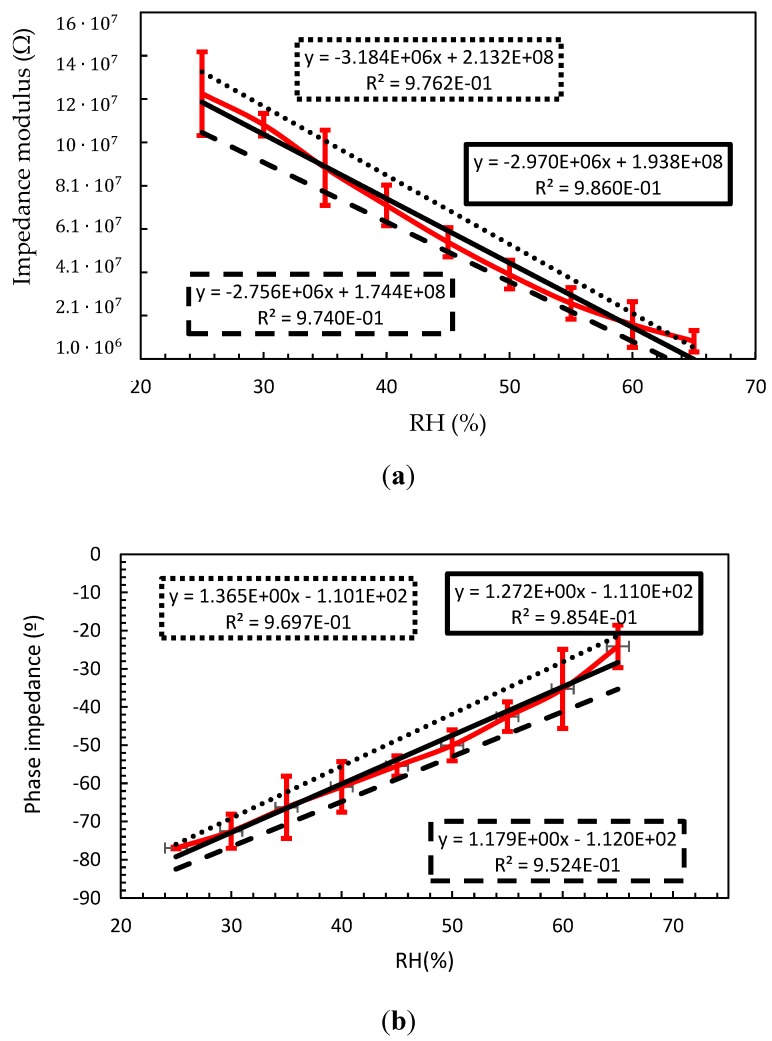
Sensor impedance at 200 Hz with a 95% of confidence interval. Red line represents the average measured impedance with 95% confidence interval error, continuous black line represents the linear regression for the average value, and the dotted and dashed lines represent the linear regression for +9% confidence interval and −95% of confidence interval, respectively. The linear regression equations are also shown in the graph. (**a**) impedance modulus (**b**) impedance phase.

Figure 5 shows the measured module and phase impedance at 200 Hz, where the red line represents the average measured impedance with a 95% confidence interval error, continuous black line depicts the linear regression for the average value, and the dotted line and dashed lines represent the linear regression for +9% confidence interval and 95% confidence interval, respectively. The linear regression equations are also shown in the graph. From this data, a linear dependence behavior is observed with the moisture. However, due to the manufacturing variability, the static sensor characteristic shows a clear variability. Table 1 summarizes the dispersion measured on the sensitivity and zero shift parameter of the sensor impedance. In particular, the sensitivity of the sensor impedance module has a value of 2.97 MΩ/%RH ± 7%, meanwhile the average zero shift is 193.8 MΩ ± 10%. Meanwhile, the value of the sensor impedance phase achieves a sensitivity value of 1.272°/%RH ± 7.3% and the zero shift a value of −111° ± 0.9%. 

**Figure 6:**
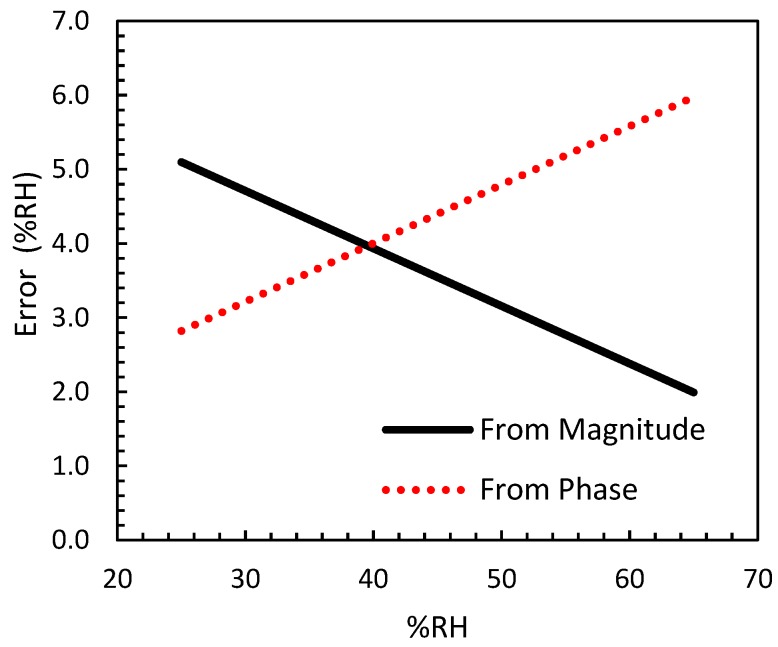
Error of relative humidity at 200 Hz due to the manufacturing variability. The errors were obtained from the magnitude and phase impedance measurement of the sensor.

From the previous dispersion values, it is possible to determine the expected error on module and phase impedance due to manufacturing variability. The results are depicted in Figure 6. A maximum error lower than 6% on the moisture measurement was obtained. It should be noted that the error decreased with the moisture when the impedance modulus was measured. Meanwhile the phase error increased with the moisture. According with this behavior, and in order to reduce the error up 4% on the moisture measurement, for moisture values lower than 40% RH, the impedance phase should be used. However, for higher moisture values, the moisture value should be obtained from the impedance module measurement.

### 3.2. Washing Cycles

**Figure 7:**
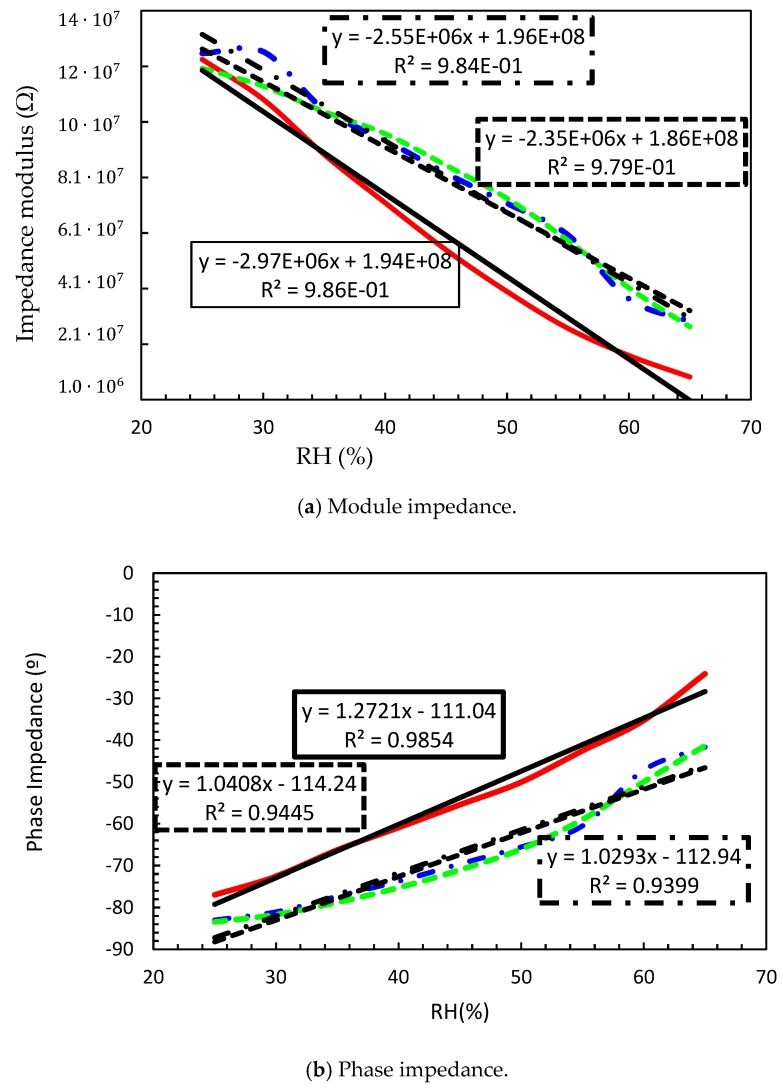
Effect of washing cycles on the impedance at 200Hz. Before washing (continues line), after one washing cycle (dot-dash line), and after two washing cycle (dash line). The linear regression for each case and the equations are also show.

In order to assure the success of e-textiles in real applications, these products should guarantee their functionality after the washing process. At this point, the electrical behavior of the proposed interdigitated textile sensor was evaluated after applying a washing process. Figure 7 shows the sensor impedance module and impedance phase without washing (continuous line), after applying one conventional washing cycles (dash-dot line) and after applying two washing cycles (dash-line). The linear regression for each case and the corresponding equation are also shown. 

It was observed that after applying the washing cycles, the impedance module increased for all moisture values whereas the impedance phase was reduced. This behavior points out that after washing cycles the capacitance behavior of the proposed sensor decreased, meanwhile the resistance increased. A small significant difference was observed between one and two washing cycles. This fact is explained by the commercial fabrics’ manufacturing process. In order to guarantee the distributions, the textiles are subjected to a specific antibacterial treatment. After washing, this treatment disappears, and this explains the reason of the similar electrical impedance after one and two washing cycles. 

Table 2 summarizes the impact of washing cycles on sensor behavior. A clear difference before and after washing was observed. After the first washing, impedance module sensitivity was reduced by 14.14%, meanwhile, the zero drift was shifted just 1%. However, after the second washing cycle, only an additional 7.8% of reduction was observed, which represents a reduction of 20.88% with regard to unwashed samples. With respect to the impedance phase, almost no differences were observed between one or two washings. After washing the sensitivity was reduced between 18–19% and the offset about 2% in both cases. As previously mentioned, the used fabric has an antibacterial treatment that modifies its dielectric properties. In fact, this antibacterial treatment consists of an increase in the electrical conductivity of the fabric. Therefore, before washing, the treatment makes the sensor more conductive but, when the sensor was washed, this treatment was deleted, decreasing the conductivity of the fabric and therefore increasing the sensor impedance.

## 4. Conclusions

In this work, an interdigitated embroidered textile sensor was proposed and the manufacturing variability and washing impact were characterized. The sensors were embroidered over a cotton substrate with a commercial Shieldex 117/17 dtex 2 yarn. The measured results demonstrate experimentally the usefulness of the proposed sensors at the kHz range to develop wearable applications over textile materials for moisture measurement. Due to the manufacturing variability process, an error lower that 6% on the RH measurement was obtained. However, this error can be reduced up to 4% when both the module and phase impedance of the sensor are measured. The washing process of the textile sensor also impacted the electrical behavior, mainly after the first washing cycle, when the treatment of the fabrics disappeared, this effect was mainly observed as a reduction on the sensor sensitivity. In any case, the devices kept some of their sensing capabilities.

## Figures and Tables

**Table 1:** Sensor impedance properties with process variability for 95% interval of confidence.

Impedance Modulus				Impedance Phase			
	min	mean	max		min	mean	max
**Sensitivity** $\left(\frac{M\Omega }{\%RH}\right)$	−3.184	−2.97	−2.756	**Sensitivity** $\left(\frac{{}^{\circ}}{\%RH}\right)$	1.179	1.272	1.365
**Zero shift**(MΩ)	174.4	193.8	213.2	**Zero shift** (°)	−112	−111	−110

**Table 2:** Relation between the parameters measured and the relative humidity.

	Module				Phase			
Impedance	$\mathrm{Sensitivity}\;\left(\frac{M\Omega }{\%RH}\right)\;$	ΔS%	Zero Shift MΩ	ΔZs %	$\mathrm{Sensitivity}\;\left(\frac{{}^{\circ}}{\%RH}\right)\;$	ΔS %	Zero Shift °	ΔZs %
No-wash	−2.97		194		1.272		−111.04	
1 wash	−2.55	−14.14	196	1.03%	1.029	−19.1	−112.94	1.71
2 washes	−2.35	−20.88	186	4.12%	1.041	−18.16	−114.24	2.88
